# PTEN defects in cancer, from gene to protein molecular causes and therapeutic targets

**DOI:** 10.1007/s12672-025-03465-4

**Published:** 2025-08-28

**Authors:** Ghaith Khasarah, Darina Al Nabilsi, Odai Madani, Farah Khazem, Ranwa Alsayed

**Affiliations:** Faculty of Pharmacy, Damascus University, Damascus, Syria

**Keywords:** PTEN, PTEN and cancer, PI3K/AKT pathway, *MiRNA*, *LncRNA*

## Abstract

Recently, cancer has become a leading cause of death worldwide, prompting increased research to understand the pathways involved in cancer development and to identify solutions for its treatment. The PI3K/AKT pathway has garnered significant attention because of its involvement in promoting cell proliferation and inhibiting programmed cell death. The protein phosphatase and tensin homolog on chromosome 10 (PTEN) plays a crucial role in inhibiting this pathway, thereby limiting uncontrolled cell proliferation. *PTEN* gene expression is strictly regulated at the transcriptional, posttranscriptional, and posttranslational levels, and recent research has focused on PTEN due to its reduced levels in cancer cells. This review aims to provide a deep understanding of the PTEN protein from structural and regulatory perspectives, its mutated forms, and its interactions with the occurrence of various malignant tumors to summarize the recent work performed to combat cancers via molecular strategies to enhance PTEN.

## Introduction

The phosphatase and tensin homolog on chromosome 10 (PTEN) gene is one of the most important tumor suppressor genes and is located on the long arm of chromosome 10 at position 10q23.31 [1]. This gene encodes a dual-specificity phosphatase protein (lipid and protein phosphatase) that plays a critical role in regulating several vital cellular pathways.

The mutational status of PTEN is important in the development of various cancers, including liver, breast, lung, and prostate cancers [1], where its loss or inactivation is associated with increased activity of the PI3K/AKT pathway, leading to increased cell proliferation and the inhibition of programmed cell death (apoptosis) [2]. PTEN suppresses this pathway through its phosphatase activity by dephosphorylating PIP3, converting it to PIP2, which prevents the activation of AKT and subsequently inhibits the mTOR pathway. Furthermore, PTEN is responsible for regulating cellular translation, cell growth and controlling cell division, migration, metabolism, and stem cell renewal [3]. PTEN also plays a crucial role in cell cycle regulation by enhancing the recruitment of the protein p27 to the E/CDK2 complex [4], thereby preventing cells from entering the S phase and limiting their uncontrolled proliferation [4].

Its expression is tightly regulated by noncoding RNAs such as miRNAs, which inhibit PTEN mRNA translation. Conversely, molecules such as PTENP1 and lncRNAs act as competitive endogenous RNAs (ceRNAs), competing with PTEN mRNA for miRNA binding and thereby restoring PTEN expression.

In this review, we discuss the molecular structure and regulation of PTEN, the mechanisms leading to its dysregulation in various cancers, and therapeutic approaches that target this critical pathway, particularly those focused on restoring or enhancing PTEN function in tumors, whether by exporting vesicles out of a cell or by finding ways to repair genetic defects via CRISPR technology.

We aimed to cover the most important molecular regulation and its therapeutic application in our review.

## PTEN gene and its structure

**Fig. 1:**
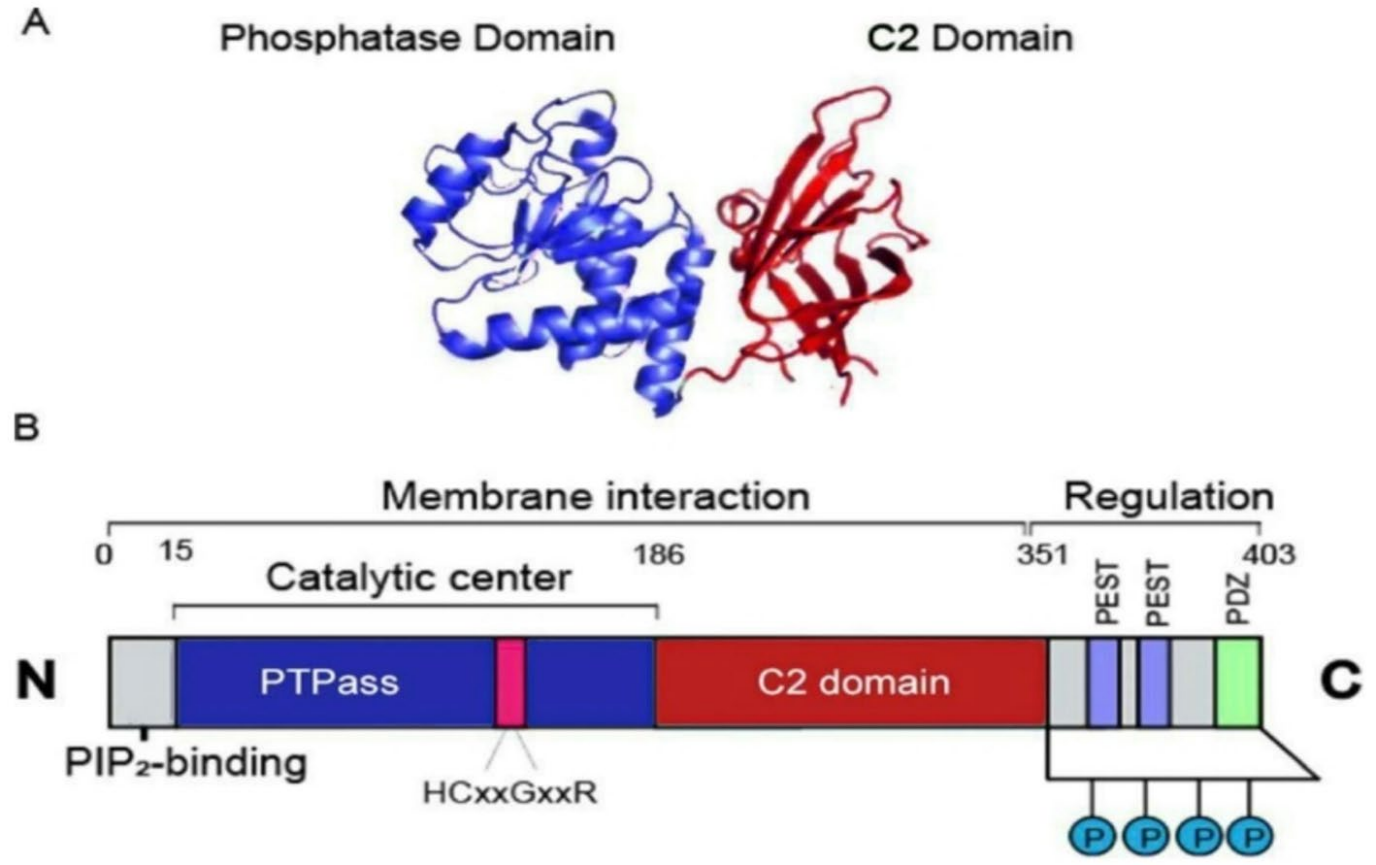
PTEN protein structure. Section (A) shows the structure of the PTEN domain (the phosphatase domain is shown in blue, and the C2 structural domain is shown in red), and section (B) shows that PTEN consists of 403 amino acids, the three domains of PTEN, and the catalytic center

The *PTEN* gene is defined as a tumor suppressor gene that is mutated in many cancers at a high frequency; it is located on chromosome 10q23.31 and extends over 105 kb with nine exons [1]. The protein encoded by this gene is phosphatidylinositol-3,4,5- trisphosphate 3-phosphatase [5], which has been shown through amino acid sequencing to belong to the tyrosine phosphatase family [1]. A distinctive feature of this protein is the wide opening in the depth of the catalytic pocket, allowing tyrosine and serine/threonine residues to access cysteine residues [1]. Structurally, the protein consists of two folded globular domains, the C2 domain and the phosphatase domain Fig. 1A, with three disordered protein segments (PDZ-binding domains, C‐tails, and PBD‐domains) [1, 6]. These segments are essential for membrane binding and allosteric regulation, which controls protein function and is critical for generating PIP2, as the PIP2 binding site and adjacent cytoplasmic localization signal are located in the N-terminal domain of the protein [6] see Fig. 1B.

Note that here are two areas (PESTs) this sequence should be mentioned in the text above! associated with PTEN. PEST sequences are associated with the expression of PTEN [6].

However, PTEN contains two PEST sequences situated in the C-terminal region, which are likely involved in targeting the protein, as deletions here decrease PTEN stability [7]. The phosphatasedomain of PTEN contains critical residues such as cysteine (C124), where mutations (C124S) abolish enzymatic activity, leading to hyperactivation of the PI3K/AKT pathway [8].

Most mutations in the protein occur in the C-terminal C2 domain corresponding to exons 6, 7, and 8, as well as in the tail sequence corresponding to exon 9, which encodes the phosphorylation sites for tyrosine kinases that are also essential for protein function [1], whereas other regions remain unaffected by mutations.

In this section, the gene, the structure of its encoded protein, its regulatory regions, and associated mutations were examined, establishing a foundation for understanding its regulatory mechanisms and exploring potential therapeutic strategies.

## PI3K/AKT/PTEN pathway

The PI3K family is defined as membrane-associated lipid kinases that can phosphorylate the 3’ hydroxyl group of phosphatidylinositol and phosphoinositides. PI3Ks are divided into three classes (class I, class II, and class III) [9] on the basis of their structures and functions. Class I PI3Ks are the most distinctive and are generally associated with extracellular stimuli and signaling, responding to the activation of cell surface receptors, whereas classes II and III are involved in membrane transport [10]. The PI3K signaling network regulates cell growth, division, migration, and survival, but abnormal regulation of this signaling pathway can lead to cellular dysfunction and often to cancer development [11].

**Fig. 2:**
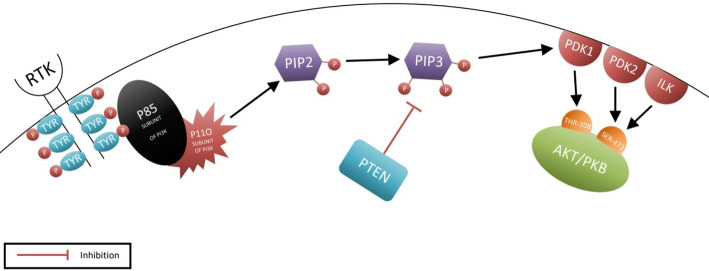
The tumor suppressor PTEN attenuates PI3K/AKT signaling via PIP3-to-PIP2 conversion. Illustrates the mechanism by which the PI3K/AKT pathway is activated within the cell through a series of signals that begin with the binding of the ligand to the receptor tyrosine kinases and end with the activation of AKT, leading to tumor growth induction, and how PTEN intervenes to inhibit this pathway and counteract tumor development. This image was generated by drawing software to provide a brief overview of the PI3K/AKT pathway and the site of PTEN intervention

The PI3K/AKT/PTEN pathway is considered an important therapeutic target that has garnered significant attention. The proteins involved in this pathway are phosphoinositide 3-kinase (PI3K) and protein kinase B (PKB), known as AKT, while the receptors involved are receptor tyrosine kinases (*RTKs*) located in the cell membrane [12]. The mechanism begins with the binding of a ligand to the *RTK*, leading to autophosphorylation of the tyrosines present on it [12]. The p85 regulatory subunit of PI3K is attracted to these phosphorylated tyrosines, which also bind to the p110 catalytic subunit, forming the PI3K protein complex [13]. This complex converts PIP2 to PIP3 through phosphorylation. PIP3 then recruits AKT/PKB to the cell membrane, where membrane-associated kinases phosphorylate parts of AKT. 3-Phosphoinositide-dependent kinase‑1 (PDK1) functions as a pivotal upstream kinase. When receptor activation occurs, PI3K generates PIP₃ at the cell membrane, recruiting both AKT and PDK1 via their PH domains; PDK1 phosphorylates AKT at Thr308, initiating partial activation [14]; and PDK2 and integrin‑linked kinase (ILK) (the kinases responsible for phosphorylating Ser473 in the hydrophobic motif of AKT) phosphorylate serine 473 in AKT [15,16], resulting in activated AKT, which translocates from the membrane back to the cytosol and nucleus to perform various functions [12]. It is involved in the activation of several pathways, such as the *mTOR* pathway, glycogen synthase kinase-3 (GSK3), programmed cell death, and cell survival. Notably, the role of the PTEN protein is significant in regulating or inhibiting this pathway, as it converts PIP3 back to PIP2, preventing the activation of AKT and its functions *(*Fig. 2*)* [12], highlighting the critical therapeutic role of PTEN in tumor suppression, given its pivotal involvement in this signaling pathway.

Dysregulation of the PI3K/AKT pathway is involved in many human diseases, including cancer, diabetes, cardiovascular diseases, and neurological disorders [17]. In cancer, mutations that increase PI3K kinase activity have been identified. Additionally, PTEN is often mutated or lost in human tumors, and activating mutations in AKT have also been found [17].

Crucially, the activity of PTEN must be maintained to fulfill its role in suppressing this pathway, thereby inhibiting tumor progression.

While numerous pathways are implicated in PTEN-related carcinogenesis, this review focused on this particular pathway because of its critical regulatory and therapeutic relevance.

In addition to its lipid phosphatase activity, PTEN acts as a dual-specificity phosphatase with the ability to dephosphorylate protein substrates. Notably, loss of this protein phosphatase activity has been linked to various diseases, including defects in neural stem cell differentiation [18].

## PTEN: its expression, regulation and mutations

As previously mentioned, PTEN is a tumor suppressor, and any defect in it leads to the development of various tumors. Understanding the cause of this defect can pave the way for potential therapeutic strategies, such as regulatory pathways involving various molecules, *such as miRNAs*, *PTENP1* and long noncoding RNAs (lncRNAs). Importantly, there are numerous molecules that regulate PTEN, some of which have not yet been therapeutically exploited.

Alternatively, the defect may result from multiple mutations that are important for understanding the underlying causes of certain PTEN-related tumors or can be used therapeutically, which will be discussed later.

### PTEN expression by mirna, PTENP1 and LncRNA

Initially, protein levels are regulated at the transcriptional and posttranscriptional levels, in which miRNAs play pivotal regulatory roles [2], serving as key molecular agents that directly target PTEN. In contrast, PTENP1 and LncRNA exert their regulatory effects on PTEN through indirect mechanisms.

#### PTEN miRNA-mediated regulation

*miRNAs*, or small noncoding RNA molecules, regulate gene expression at the transcriptional and translational levels [5], and miRNAs can regulate the expression of PTEN either by directly targeting PTEN mRNA (for example, miR-21, miR-221/222, and miR-301a) or by inducing hypomethylation of the PTEN promoter (such as miR-29, miR-101, and miR-185) [19]. Recent studies have shown their involvement in the occurrence or development of various diseases, particularly malignant tumors, as many of them are found in fragile sites of the human genome or in regions that are amplified or deleted in cancers [20]. As previously mentioned, PTEN plays an important role in tumor suppression and promoting programmed cell death, and its levels are decreased in the cancerous tissues of mouse models because specific *miRNA*s target it by acting as sponges for the PTEN mRNA [2]. *miR*-*22* plays a significant role in the initiation and progression of both breast and prostate cancers by suppressing PTEN and significantly affecting the tumor microenvironment [21]. Similarly, *miR*-*130* plays a role in tumor induction by reducing PTEN expression in various cancers, such as bladder cancer, invasive breast cancer, kidney cancer, gastric cancer, gliomas, lung cancer, and colon cancer. However, other studies have shown that the overexpression of *miR*-*130* plays a critical role in increasing *PTEN* levels and promoting programmed cell death in non-small cell lung cancer [22]. Additionally, miR-17, miR-19b, and miR-20a play key roles in tumor induction and the inhibition of PTEN in hepatocellular carcinoma [23]. The same applies to miR-200a, miR-200b, and miR-200c in endometrial cancer [24], as well as miR-17-92, which plays a significant role in promoting cancers by inhibiting PTEN in various types such as ovarian, breast, and colorectal cancers [25]. Furthermore, miR-214 is also involved in breast and prostate cancers [26]. Notably, many types of miRNAs negatively regulate PTEN, and we discuss them in detail later, as they are targeted in novel cancer therapeutic approaches. In summary, miRNAs play crucial roles in the negative regulation of *PTEN* levels, thereby promoting cancer development by activating the PI3K/AKT pathway.

#### Identifying PTENP1 forms and how they regulate the abundance of PTEN

*PTENP1* is a gene that is highly similar to *PTEN* in sequence, producing two types of transcripts [27]. *The* sense transcript *PTENP1-S* is a gene variant resembling long noncoding RNA (*lncRNA*) but has high similarity to the *PTEN* sequence, making it a suitable target for *PTEN* inhibitors by acting as a sponge and inhibiting the action of *miRNAs* [28].

**Fig. 3:**
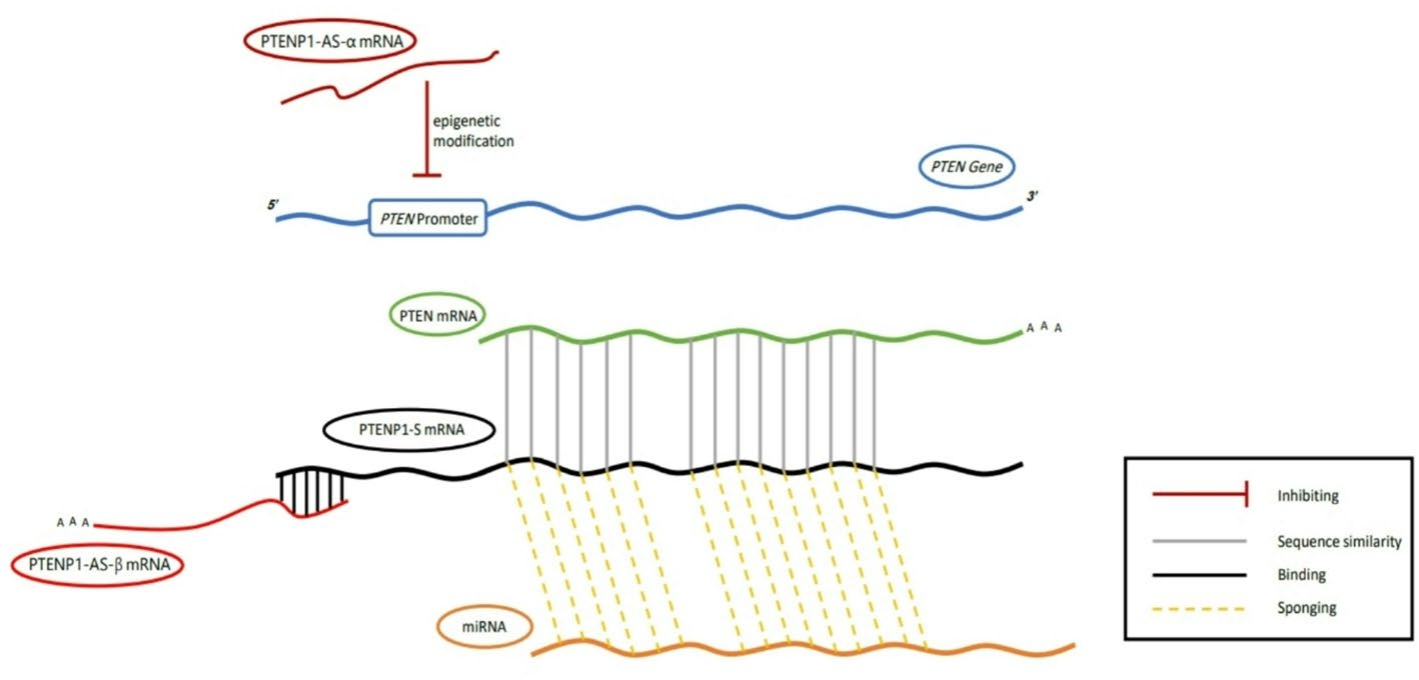
Roles of PTENP1 transcripts in regulating PTEN levels. The regulatory functions of PTENP1 transcripts are distinct: PTENP1-AS-α suppresses PTEN expression by recruiting epigenetic modifiers to the promoter region, whereas PTENP1-AS-β stabilizes the PTENP1-S transcript through polyadenylation. This stabilization enables PTENP1-S to act as a competitive endogenous RNA (ceRNA), sequestering miRNAs and thereby safeguarding PTEN mRNA from degradation. Together, these mechanisms fine-tune PTEN levels through coordinated transcriptional and posttranscriptional regulation

The antisense transcript consists of the isoforms *PTENP1-AS-α* and *PTENP1-AS*-β, which are structurally similar and functionally independent. *PTENP1-AS-α* resides at the *PTEN* promoter region and recruits epigenetic modifiers, resulting in a negative effect on *PTEN* transcription [29]. Moreover, *PTENP1-AS-β* associates with the *PTEN*1-S transcript, which lacks a poly A tail (contributing to mRNA stability and efficiently affecting translation), ensuring the stability of the *PTENP1*-*S* transcript by forming a *PTENP1*-*S*-*PTENP1-AS-β complex* that is then released into the cytoplasm to act as a *miRNA* sponge [29, 30] (Fig. 3).

This finding was confirmed by studies in which both *PTEN* and *PTENP1* were silenced in DU145 prostate cancer cells [31]. Inhibition of *PTENP1-S* suppressed *PTEN* expression, whereas silencing *PTENP1-AS-α* and *PTENP1-AS-β* reduced the regulation of both *PTEN* and *PTENP1* [31].

Hypermethylation of the *PTENP1* promoter results in the deletion or silencing of the *PTENP1* protein, leading to the loss or decrease in the expression of *PTEN* and, subsequently, *PTENP1* [32]. Other studies have shown that the abundance of *PTENP1* is associated with the abundance of *PTEN*, as increases in both *PTENP1-S* and *PTENP1* lead to decreases in *PTENP1-AS-α*, whereas increases in *PTEN* lead to increases in *PTENP1-AS-β* [29].

Unsurprisingly, the *PTENP1-S* transcript has become a focal point after all that has been discussed. In addition, its role as a tumor suppressor is similar to that of *PTEN* but independent [33]. Given that these studies focused on the existence of low levels of *PTENP1* in cancer and its activity as a tumor suppressor, we expect it to be a promising candidate biomarker for the early detection of cancer in the future, as its reduced expression may predict poor prognosis for various tumors [34].

#### PTEN LncRNA-mediated regulation

Long noncoding RNAs (lncRNAs) are a type of RNA molecule that is approximately 200 nucleotides long [35] and plays crucial roles in modulating gene expression at both the transcriptional and posttranscriptional levels [36]. It also plays an important role in modifying the function of *PTEN* [37]. Numerous studies have shown that dysfunction in *lncRNAs* is associated with tumor progression and the resistance of cancer cells to treatment [38]. Among other roles, many act as regulators of the important PTEN/PI3K pathway in the cell cycle of cancer cells by acting as sponges of the PTEN mRNA [38, 39].

Some *lncRNAs* function as inhibitors of the PI3K/AKT pathway [40] by interacting with microproteins and noncoding RNAs within the pathway [41]. These interactions stabilize the key components of this pathway, negatively affecting cancer cell growth [36]. Alternatively, *lncRNAs* can act as molecular sponges by binding to miRNAs, preventing them from binding to PTEN mRNA, which increases the expression of PTEN and consequently inhibits tumor growth [35].

In addition, some *lncRNAs* increase the expression of PI3K [42], which is considered to promote cancer cell growth. This occurs through the abnormal and inappropriate regulation of *long noncoding RNAs (lncRNAs)* with normal cellular activities [43]. Thus, *lncRNAs* play a significant role in the context of cancer cells, either by inhibiting or promoting their proliferation.

### PTEN and PTENP1 mutations and their variants in various cancers

Disruptions in the PTEN gene, such as deletion or mutation, lead to the occurrence of various cancers [2]. Additionally, the deletion of *PTEN* leads to a decreased immune response against tumors [2]. Tumors lacking PTEN are characterized by reduced levels of helper T cells, natural killer cells, and cytotoxic lymphocytes [44], along with increased levels of inflammatory cytokines that promote cancer, particularly C-C motif chemokine ligand 2 *(CCL2)*, and an increase in immune-suppressive cells such as myeloid-derived suppressor cells (MDSCs) and regulatory T cells (Tregs) [1].

**Fig. 4:**
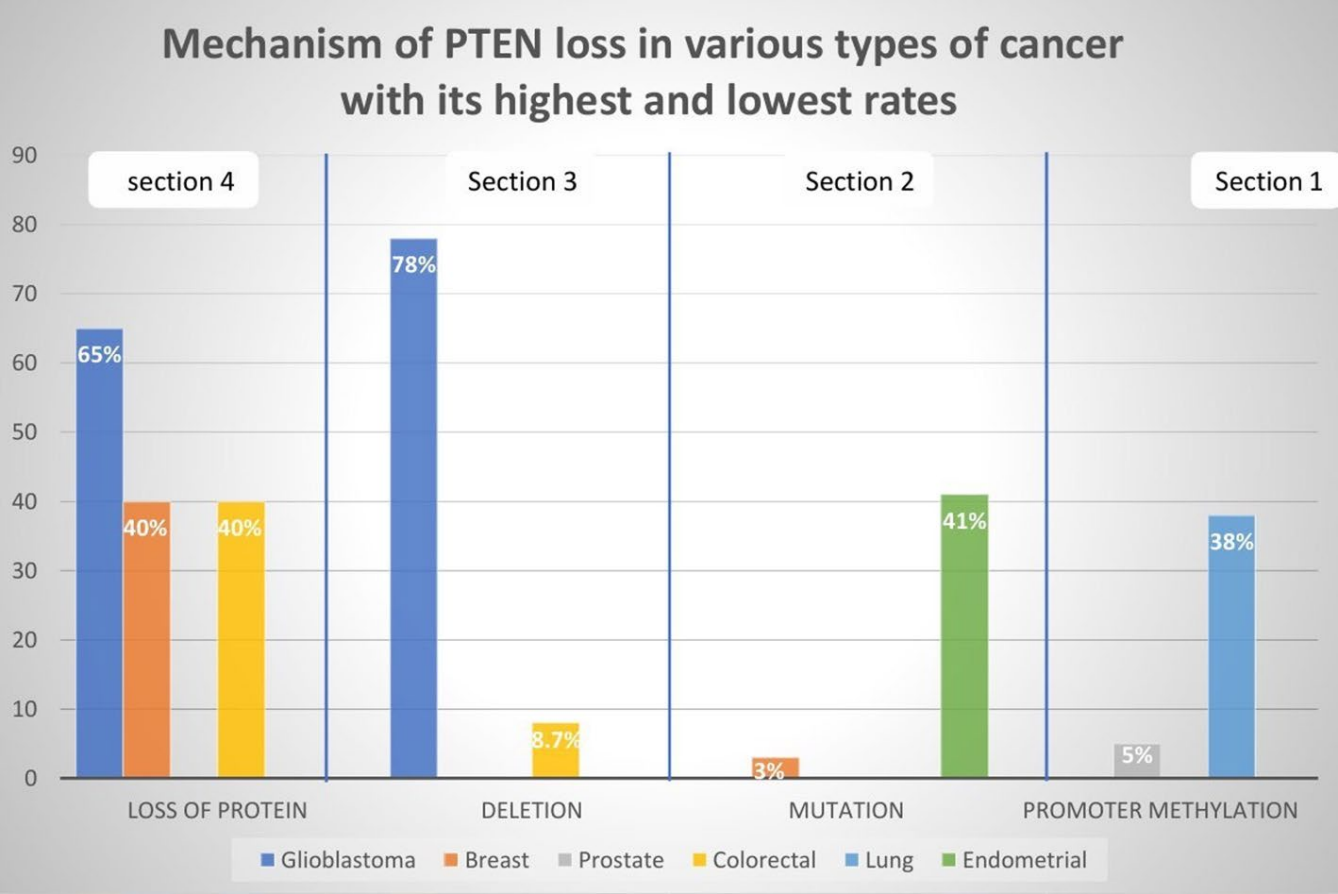
Mechanism of PTEN loss in various types of cancer with the highest and lowest rates. Inactivation of PTEN in cancer shows distinct patterns across tumor types. For example, through a protein promoter methylation mechanism, the highest percentage of lung cancer cases is 38% [45], and the lowest percentage of prostate cancer cases is 5% [46] (Sect. 1). Mutations are highest in endometrial cancer (41%) [47] and lowest in breast cancer (3%) [48] (Sect. 2). On the other hand, glioblastoma has the highest percentage of protein deletions, at 78% [49], whereas colorectal cancer has the lowest percentage according to the previous mechanism, at a rate of 8.7% [50] (Sect. 3). Finally, through the protein loss mechanism, we find that glioblastoma accounts for the highest percentage at 65% [51], whereas colorectal [50] and breast cancer [45] account for the lowest percentage at 40% (Sect. 4). These tumor-specific variations highlight the diverse molecular mechanisms underlying PTEN pathway disruption in different malignancies

Mutations in the PTEN gene lead to various types of cancers, whether these mutations are point mutations, complete deletions, or allele-specific deletions [2], or through epigenetic silencing (hypermethylation of the *PTEN* promoter) [2], as shown in Fig. 4.

Additionally, germline mutations in the PTEN protein lead to four syndromes that share the commonality of developing from benign hamartomatous tumors (nodules of tissue resembling a tumor that differ from the surrounding tissues) and are classified under the term *PTEN* hamartoma tumor syndrome (*PHTS*) [52]. These include Cowden syndrome [53], Bannayan–Riley–Ruvalcaba syndrome (*BRRS*), Proteus syndrome, and Proteus-like syndrome. Patients with these syndromes are at risk of developing malignant tumors, such as breast cancer (approximately 80%), thyroid cancer (approximately 30%), renal cell carcinoma (approximately 34%), endometrial cancer (approximately 28%) [54], and colorectal cancer (approximately 9%) [54, 55].

Although complete loss-of-function mutations in the PTEN gene are often lethal or result in severe syndromic conditions such as PTEN hamartoma tumor syndrome (*PHTS*), even a partial reduction in PTEN function, most commonly through haploinsufficiency, can have substantial clinical consequences [56]. When one allele is functionally inactivated, the remaining wild-type allele may be insufficient to maintain proper tumor suppressor activity [56]. This suboptimal PTEN dosage has been associated with a range of pathological outcomes, including abnormal brain development [57]; autism spectrum–like behavioral manifestations [58]; increased susceptibility to breast, prostate, and thyroid cancers [59]; and disturbances in glucose metabolism and insulin signaling pathways [60].

*PTENP1-S* is methylated in endometrial cancer and hyperplasia but not in normal tissue [61]. Additionally, a recent study revealed that methylation of *PTENP1* leads to increased expression of *PTENP1-S* in normal endometrial tissues and endometrial hyperplasia from women aged 45 years and over and/or women approaching, or in, menopause [62]. A recent study conducted on melanoma cells revealed that increased expression of *PTENP1-AS* led to the induction of cell resistance to *BRAF* inhibitors, which may be due to the employment of epigenetic modifiers in the *PTEN* promoter region, resulting in decreased expression of PTEN [63], and in samples from stage III melanoma patients, high levels of *PTENP1-AS* were noted and associated with decreased survival [63].

From the above, some close correlations have been clarified. Elevated levels of both *PTEN* and *PTENP1-S* were notable in certain malignant tumors, such as osteosarcoma cell lines [64] and melanoma cell lines [63], whereas decreased levels of *PTEN* and *PTENP1-S* were observed in other samples, such as prostate cancer tissues and gastric cancer cell lines [17, 65]. Furthermore, decreases in both *PTENP1-S* and *PTENP1-AS* levels were noted in kidney, breast, cervical, and bone cancers, as well as in *HEK-293T*, *MCF-7*, *HeLa* and *U-2OS* cells [27].

## PTEN regulation of cancer via exracellular vesicles

The tumor suppression PTEN plays a pivotal role in modulating cancer progression. Emerging evidence suggests that PTEN not only functions intracellularly but also exerts its effects via extracellular vesicles (EVs), including exosomes, influencing both tumor and stromal cell behavior.

### PTEN secretion in EVs influence neighboring cells

PTEN can be selectively packaged into exosomes via the Ndfip/Nedd4-dependent ubiquitin ligase system and secreted by cancer cells (Ndfip is an adaptor protein for members of the Nedd4 family of E3 ubiquitin ligase; it enables Nedd4 to ubiquitinate targets such as PTEN, regulating their trafficking and secretion (including via exosomes)) [66, 67]. Importantly, PTEN delivered through EVs remains functionally active; upon uptake by PTEN-deficient recipient cells, it suppresses the PI3K/AKT signaling cascade, leading to reduced cyclin D1 expression (cyclin D1 is the regulatory protein that controls the progression of cells through the G1 phase of the cell cycle) [68], increased p27 levels, and subsequent cell cycle arrest [66]. For example, exosomal PTEN transfer from PTEN-positive to PTEN-null prostate cancer cells has been shown to induce G1 phase arrest, indicating its tumor-suppressive intracellular communication capacity [66]. EV-mediated PTEN transfer serves as paracrine tumor-suppressive mechanism in the tumor microenvironment.

As a result, we found that EV-mediated PTEN transfer acts as a tumor-suppressive intercellular signal, especially in PTEN-deficient recipient cells, which may have therapeutic implications, as it opens the possibility of engineering similar vesicles for future clinical applications.

### PTEN loss enhances EV secretion and promotes tumor progression

#### Lysosome-EV trafficking in cholangiocarcinoma

Loss of PTEN impairs transcription factor (TFEB)-mediated lysosomal biogenesis, resulting in reduced lysosomal degradation of multivesicular bodies (MVBs) (MVBs are intracellular organelles that contain small intraluminal vesicles (ILVs)). When the MVBs fuse with the lysosomes, their ILV contents are degraded, contributing to cellular waste disposal and protein turnover. Alternatively, when MVBs fuse with the plasma membrane, they release ILVs into extracellular space as exosomes, which mediate intercellular communication by transferring bioactive molecules such as proteins, RNAs, and lipids. In cancer, deregulation of MVB formation and trafficking can promote tumor progression by enhancing exosome-mediated signaling, immune evasion, and metastasis [69, 70] and, consequently, increasing EV secretion [71]. This elevation in EV output enhances tumorcell proliferation, migration, and metastasis in cholangiocarcinoma models. Notably, pharmacological activation of TFEB restores lysosomal function and mitigates the metastatic phenotype, highlighting the role of the PTEN-TFEB-Lysosome-EV axis in tumor progression [71].

#### Immune suppression via PD-L1 loaded EVs in glioma

In glioblastoma, PTEN deficiency increases PI3K/AKT pathway activation, which in turn enhances the biologenesis of EVs enriched with PD-L1 (PD-L1 is expressed on tumor cells and inhibits T-cells function by binding to PD-L1, Leading to apoptosis or functional exhaustion of T cells) [72, 73]. These PD-L1-positive EVs downregulate T-cell receptor signaling in recipient immune cells, promoting immune evasion and establishing an immunosuppressive TME [72]. Eventually, PTEN loss leads to dysregulation of EV biogenesis and cargo loading, favoring oncogenic signaling, immune escape, and metastatic behavior; therefore, we assume that EVs can be considered promising biomarkers, offering valuable insights into tumor behavior and opening new avenues for therapeutic strategies, such as targeting EV biogenesis pathways, as a potential approach to cancer treatment.

### EV-carried RNAs modulate PTEN expression in the tumor microenvironment

#### Tumor-derived MiRNAs downregulate PTEN in macrophages

EVs released by breast cancer cells may contain miRNAs such as miR-106b-5p and miR-18a-5p, which suppress PTEN expression in macrophages [74]. This suppression leads to activation of the PI3K/AKT and JAK/STAT pathways (the JAK/STAT pathway is a critical cellular signaling mechanism that transmits signals from cytokine receptors on the cell surface directly to the nucleus, where it regulates gene expression). This pathway plays a key role in controlling various cellular processes, such as growth, differentiation, immune responses, and survival.

Dysregulation of the JAK/STAT pathway has been implicated in various diseases, including cancers, autoimmune disorders, and chronic inflammatory conditions [75], increases PD-L1 expression, and skews macrophages toward an M2-like phenotype, supporting tumor growth [74].

#### EV-delivered noncoding RNAs enhance PTEN expression

Conversely, some EVs deliver lncRNAs such as PTENP1, which function as competing endogenous RNAs (ceRNAs), sponging miR-17 and thereby restoring PTEN expression in recipient bladder cancer cells. This restoration of PTEN expression results in decreased tumor proliferation and invasion both in vitro and in vivo [76].

#### Parasitic EVs suppress PTEN and promote tumor growth

EVs released from the liver fluke Clonorchis sinensis contain miR-96-5p, which downregulates PTEN in host cholangiocytes. This repression confers resistance to ferroptosis and promotes cholangiocarcinoma progression [77].

As RNA cargo in EVs, including miRNAs and lncRNAs, dynamically modulates PTEN levels in tumor and stromal cells, further shaping the tumor microenvironment and cancer progression, we believe that modulating the tumor microenvironment and controlling cancer progression could be achieved by targeting the PTEN-suppressive cargo within EVs, suggesting a promising therapeutic strategy.

See Table 1 [66].

**Table 1:** Summarizes how PTEN regulates cancer progression via extracellular vesicles

Mechanism	EV cargo	Effect on PTEN	Outcome in cancer	Refs.
Direct protein transfer	PTEN protein	Restores PTEN activity	Growth arrest, suppressed proliferation/metastasis	[66]
Lysosome impairment	Increased EV release	PTEN loss	Enhanced metastasis (cholangiocarcinoma)	[71]
Immunomodulatory EVs	PD‑L1	PTEN loss → AKT activation	Immune evasion (glioma)	[72]
miRNA from tumor EVs	miR‑106b‑5p, miR‑18a‑5p	Downregulates PTEN in macrophages	Pro-tumor immune microenvironment	[74]
lncRNA EV cargo	PTENP1	Sponges miR‑17, restores PTEN	Tumor suppression (bladder cancer)	[76]
Parasitic miRNA EVs	csi-miR-96-5p	Suppresses PTEN	Ferroptosis resistance, Tumor progression (cholangiocarcinoma)	[77]

On the basis of the above findings, EV-associated PTEN coulpotentially be applied in liquid biopsy by detecting PTEN inside extracellular vesicles (EVs), especially exosomes, which will offer an exciting, noninvasive approach for cancer diagnosis and monitoring through liquid biopsy. EVs are stable in bodily fluids such as plasma and urine and carry proteins, RNAs, and lipids from tumor cells that reflect their molecular state. Since PTEN (and regulatory RNAs such as PTENP1 or miR-96-5p) are actively packed into these EVs and remain functionally intact, measuring their levels in patient fluids can provide a real-time picture of tumor suppressor activity and changes in the TME [78]. Proteomic studies have shown that EVs found in plasma reflect tissue-specific protein profiles and can accurately differentiate between healthy people and cancer patients across multiple cancer types [79], and EVs have several advantages over circulating tumor DNA (ctDNA) or circulating tumor cells (CTCs), including greater stability, a variety of surface markers, and the ability to carry diverse molecular cargo for multiomics analysis [80].Therefore, we propose that analyzing the molecular content of EVs could serve as a valuable approach for tumor diagnosis and prognosis.

Additionally, EVs may offer a reliable means to monitor treatment response and provide clinically relevant insights into the patient’s overalls condition.

## Future therapeutic prospects associated with PTEN

Recent research has demonstrated a notable increase in efforts to incorporate the PTEN gene into modern therapeutic strategies, targeting mechanisms that increase PTEN levels in cancer cells both before and after transcription. Pretranscriptional approaches involve primarily targeting genes via various techniques, most notably CRISPR technology. Posttranscriptionally, these strategies include either direct methods, such as the secretion of vesicles containing PTEN mRNA to increase PTEN levels withincancer cells, or indirect methods, such as the secretion of vesicles containing long noncoding RNAs (lncRNAs) or PTENP1. These molecules act as competitive sponges that bind to miRNAs, inhibiting their activity and thereby increasing PTEN expression within the cell, ultimately suppressing the PI3K/AKT pathway.

Different therapeutic strategies can be employed depending on the tumor type and the protein expression level of PTEN. In conclusion, targeted therapies rely on an in-depth understanding of molecular mechanisms, and the development of advanced technologies is needed to improve their efficacy and expand their applications.

### Gene targeting strategy via CRISPR technology

Reactivating the transcription of the PTEN protein can suppress many tumor-causing pathways or reduce drug resistance mechanisms by increasing the sensitivity of cells to treatment.

The clustered regularly interspaced short palindromic repeats (CRISPER) technology and the CRISPR-associated protein 9 system (Cas9) adapted from the immune system of streptococcus pyrogens have provided an effective way to reactive genes [81] so that the cas9 nuclease is directed to the DNA sequence by single-guide RNAs (sgRNAs), which stimulate double-stranded DNA cleavage [82].

The diversity of the CRISPER domain was expanded by mutation of the catalytic domains of Cas9 to yield a dead Cas9 (dCas9), which retains the ability to orient to a specific genomic domain by changing the sgRNA sequence without causing a break in the two DNA strands.

dCas9 can also combine with many effector domains for gene activation and release [83], on of which consists of three effector mechanical activators, the most important of which are VP64,P65, and Rta (VPR).

Thus, the dcas9-VRP system achieved unprecedented reactivation of tumor suppressor genes [84].Although dcas9/VPR can activate PTEN transcription in some tumors that cause AKT and mTOR pathway suppression [85], the use of the CRIPR/dCAS9 system to activate PTEN has been particularly effective in the melanoma cell line SK-MEL-28 and the breast cancer cell line SUM159,where PTEN levels have been elevated and tumorigenic pathways such as the PI3K/AKT/mTOR pathway have been inhibited [85]. The use of the dCad8/VPR system could pave the way for the treatment of mant types of cancers that suffer from PTEN loss mutation at the genetic level.

### Restoring PTEN levels via nanoparticles containing PTEN mRNA

Logically, one of the first ideas researchers consider is increasing PTEN levels in *PTEN*-deficient cells, such as cancer cells, by delivering PTEN mRNA via nanoparticles [86]. This reintroduces the PTEN protein into cells, thereby inhibiting the PI3K/AKT pathway and promoting apoptosis [87]. In the context of this concept, a study was conducted on a xenograft mouse model and mice with prostate cancer [88], where they were injected with an adeno-associated virus 9 (*AAV9*), a nonenveloped single-stranded DNA (*ssDNA*) virus [89], carrying PTEN mRNA to express it. As a result, the progression of prostate cancer is inhibited. Given these positive results, efforts have begun to develop methods such as biological agents for gene therapy [88], as shown in Fig. 8. Reintroducing the PTEN protein into cancer cells via mRNA delivery presents several critical challenges. One major limitation is the biological instability of mRNAs, which are highly susceptible to degradation before they reach target cells [90]. Additionally, nanoparticle delivery systems often have low efficiency in penetrating tumor tissues, particularly in solid tumors with dense extracellular matrices [91]. Another concern is the potential for immune responses, as both the mRNA and the delivery vectors may be recognized as foreign, triggering inflammation or other adverse effects [92]. Achieving cell-specific targeting remains a significant obstacle, as nonspecific delivery can harm healthy cells [93]. Furthermore, once inside the cell, there is limited control over the level and duration of PTEN protein expression, which may lead to inconsistent therapeutic outcomes [94].

### Some molecules involved in treatment plans

#### MiRNA-based therapeutic strategies

In recent years, research on *miRNA*s has increased at both the diagnostic and therapeutic levels [20]. Generally, therapeutic agents can be classified into monoclonal antibodies or large proteins. However, these agents may not fulfill the required purpose because of difficulties in reaching the target site.

On the other hand, RNA-based therapies offer a promising opportunity to achieve satisfactory therapeutic outcomes [20]. There are various delivery methods for *miRNA*s based on viruses, such as oligonucleotides, but they have high immunogenicity and toxicity, as well as size limitations. Therefore, alternative nonviral methods, such as the use of lipids, polymers, and noncellular vesicles, have been discovered [2].

##### Direct role of MiRNAs in cancer promotion

**Fig. 5:**
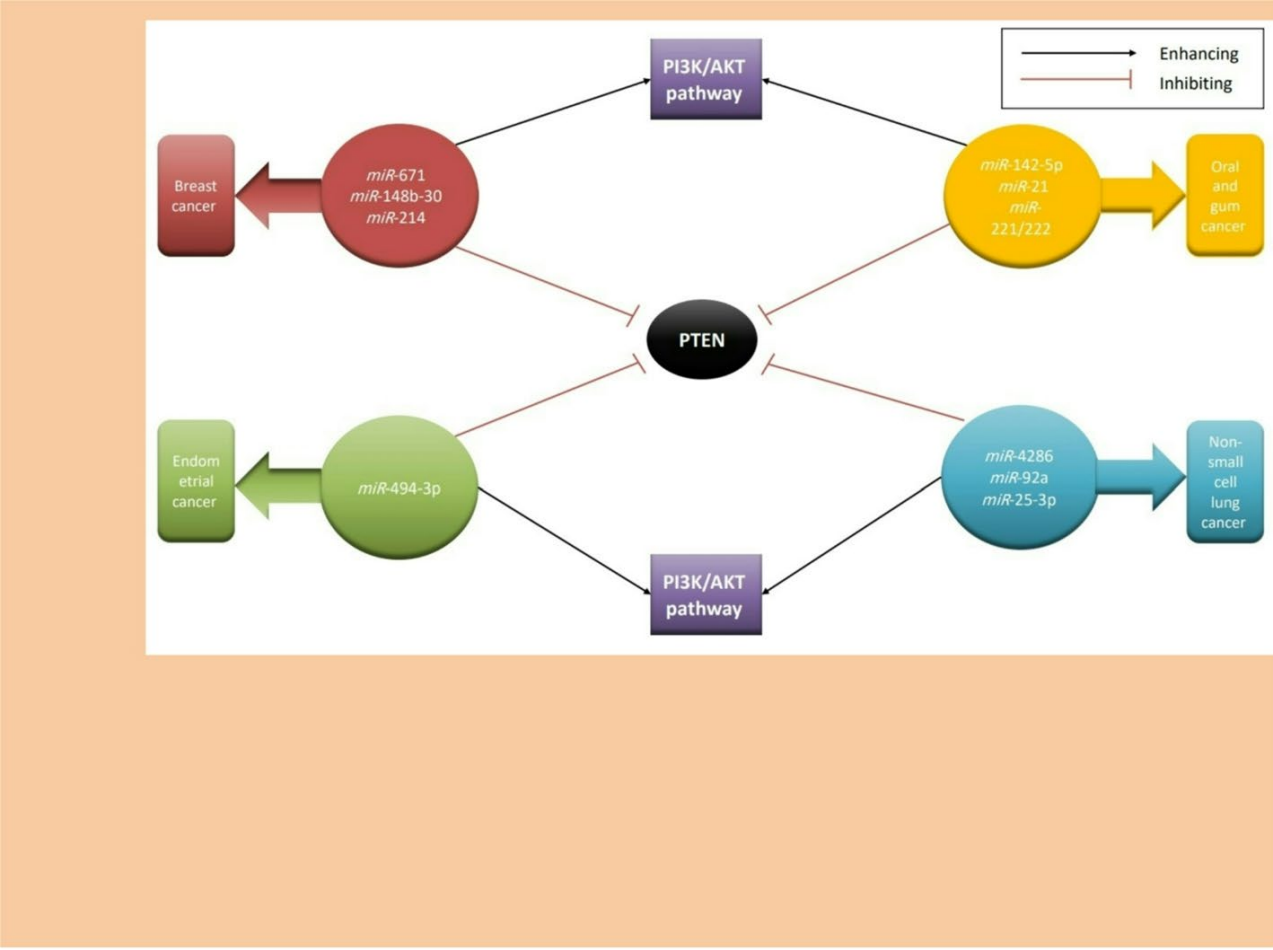
PTEN-Targeting miRNAs in multilineage carcinogenesis. Illustrates the presence of various types of miRNAs in different types of cancers and their mechanism in promoting tumor progression by inhibiting PTEN and enhancing the PI3K/AKT pathway

Returning to the core topic, which is the regulation of PTEN by *miRNA*s, studies have shown that *miRNA*s bind to the complementary regions of mRNAs located in the 3’ untranslated region (3’ UTR). When the complementarity between the *miRNA* and the 3’ UTR is complete, it leads to degradation of the protein, thereby reducing its level. Conversely, when the complementarity is incomplete, translation is suppressed [95].In summary, *miRNA*s suppress PTEN by regulating the important PI3K/AKT pathway, thus promoting cell proliferation in many kinds of cancers, such as the following:

1. Oral and gum cancer: It is one of the most common types of cancer worldwide and is characterized by difficulty in diagnosis and treatment [96]. Importantly, the PI3K/AKT/PTEN pathway plays a fundamental role in the progression of this cancer. Notably, miR-142-5p plays a crucial role in tumor development by inhibiting PTEN and activating the PI3K/AKT pathway [97]. Similarly, miR-221/222 also contribute to tumor progression through the same mechanism [98, 99]. Additionally, miR-21 is involved in this process [98, 100].
2. Non-small cell lung cancer: Many studies have demonstrated that miR-4286 [101], miR-92a [102], and miR-25-3p [103] promote tumor growth by inhibiting PTEN and activating the PI3K/AKT pathway.
3. Breast cancer: It is one of the most widespread cancers worldwide, especially among women. Multiple miRNAs are involved in the development of this cancer, particularly miR-671, which promotes tumor progression by activating the PI3K/AKT pathway and inhibiting PTEN [104]. Similarly, miR-148b-3p [105] and miR-214 [106] enhance tumor growth through the same mechanisms.
4. Endometrial cancer: Several studies have confirmed the involvement of miR-494-3p in tumor development, with elevated expression levels observed in endometrial carcinoma cells compared with normal tissues. This miRNA plays a key role in cell migration and growth by activating the PI3K/AKT pathway and inhibiting PTEN [107].

(See Table 2), as shown in Figure 5.

**Table 2:** The table shows the presence of various types of *MiRNAs* that target PTEN to form multiple cancers in different cell lines

Types of cancers	miRNAs	Refs.
Oral and gum cancer for cell lines (*SAS* and *HSC-M3*)	*miR*-142-5p	[97]
Oral and gum cancer for cell lines (*SCC15* and *SCC25*)	*miR*-21	[98, 100]
Oral and gum cancer for cell lines (*293 T*, *CAL27* and *HSC6*)	*miR*-221/222	[98, 99]
Non-small cell lung cancer in lines (*A549*, *SPC-A1*, *H1299*, *H460*, *H226* and *H1975*)	*miR*-4286	[100]
Non-small cell lung cancer in lines (*16HBE*,* A549*,* H358*,* SPC-A1* and *H1299*)	*miR*-92a	[102]
Non-small cell lung cancer in lines (*A549*,* H1299*)	*miR*-25-3p	[103]
Breast cancer in lines (*MCF7*,* T47D*,* BT474*,* MDA-MB-231* and *BALB/c nude* *mice*)	*miR*-671	[104]
Breast cancer in lines (*MCF-10 A*,* MCF-7*,* T47D*,* BT474*,* SK-BR-3*,* MDA-MB-231*, and *SUM-1315*)	*miR*-148b-30	[105]
Breast cancer	*miR*-214	[106]
Endometrial cancer	*miR*-494-3p	[61]

##### Indirect role of MiRNAs in cancer promotion

**Fig. 6:**
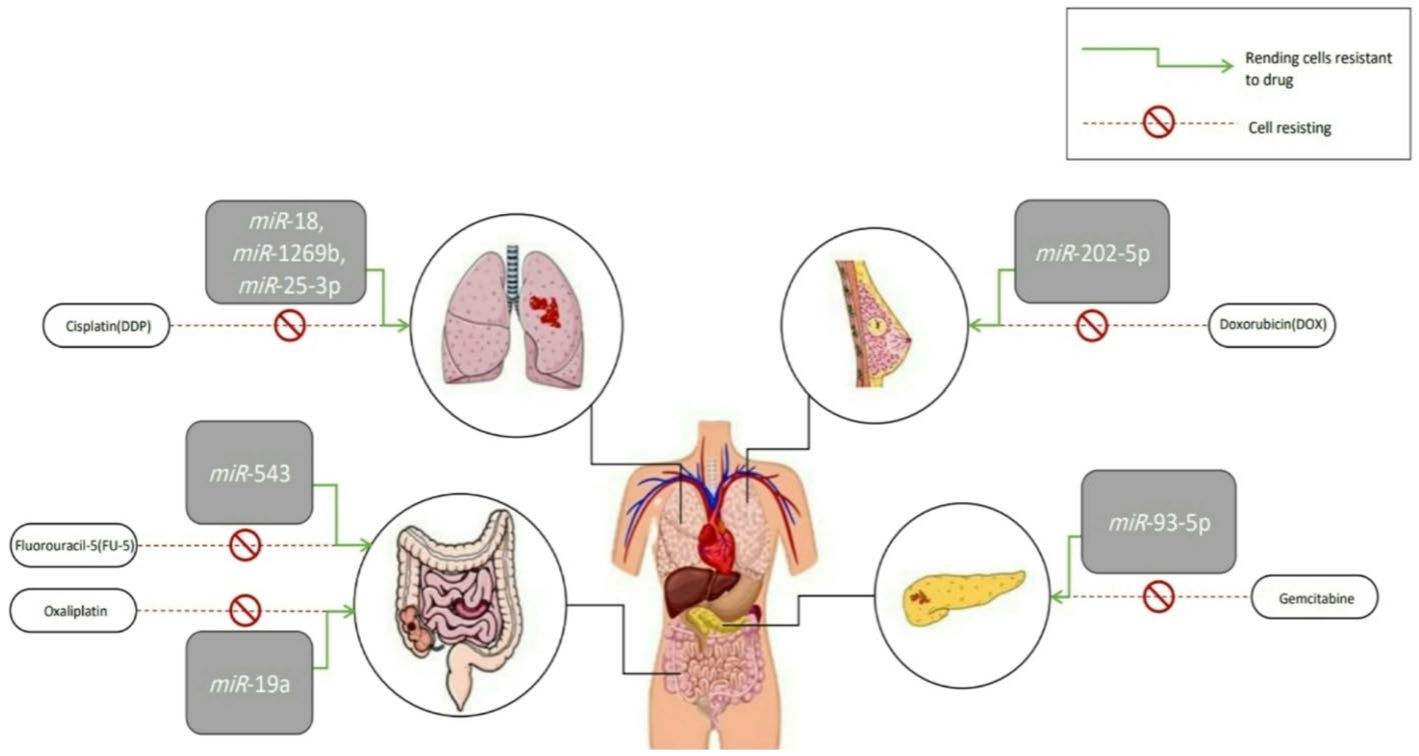
miRNAs cause chemotherapy resistance according to tumor cell origin. Illustrates one of the mechanisms by which miRNAs enhance tumor growth by rendering cancer cells resistant to various drugs that inhibit tumor development

On the other hand, recent studies have shown that *miRNAs* enhance tumor growth by making cells resistant to certain drugs via the targeting of the PTEN protein. An example of this is shown in Table 3 [108], where various therapeutic approaches have been reached by inhibiting *miRNAs* via specific sponges. Consequently, this leads to a loss of effectiveness in making cells resistant to drugs, thereby inhibiting tumor growth [108], as shown in Figure 6.

**Table 3:** The table shows examples of *MiRNAs* that promote tumor growth by making tumor cells resistant to certain drugs [108]

Non-small cell lung cancer	miR-18, miR-1269b, miR-25-3p	Making cells resistant to Cisplatin (DDP)
Breast cancer	*miR*-202-5p	Making cells resistant to Doxorubicin (DOX)
Pancreatic cancer	*miR*-93-5p	Making cells resistant to gemcitabine
Colorectal cancer (*HCT8* cell line)	*miR*-543	Making cells resistant to: Fluorouracil*-*5 (FU-5)
Colorectal cancer	*miR*-19a	Making cells resistant to oxaliplatin

Additionally, *miRNAs* can contribute to tumor development by influencing the body’s hormones. For example, *miR*-200c promotes the development of endometrial cancer by increasing estrogen levels,which play a crucial role in the progression of cancer because of its effects on both PTEN and *PTENP1*, as PTEN levels have been reported to decrease in endometrial cancer as a result of estrogen [2]. Targeting this miRNA therapeutically and studying it clinically could pave the way for a successful treatment strategy.

#### PTENP1-based therapeutic strategies

Tang et al. reported the presence of 17 *PTENP1* copies in the naked mole rat (a model organism for cancer resistance) [109]. All these genes share binding sites for *miRNAs* with *PTEN*. This similarity is likely the reason it is considered a cancer-resistant model [109]. As previously mentioned, *PTENP1-S* plays a significant role in positively regulating PTEN expression levels by competing for *miRNAs* that target PTEN to inhibit it [110]. Therefore, this approach can be utilized in a therapeutic framework to overcome certain mutations and dysregulations that lead to cancer [110]. Examples of this application include prostate [28], hepatocellular carcinoma [111], clear cell renal carcinoma [112] and oral squamous cell carcinoma [113], where the well-known *miRNA* is *miR*-21, which inhibits PTEN. *PTENP1-S* acts as a sponge, preventing the translational repression of PTEN in a competitive manner [28].

The 3’-UTR sequences of *PTEN* and *PTENP1* are similar because of the presence of *CpG* islands, which are groups of guanines and cytosines connected by a single phosphate group. Their methylation (often, cytosine is converted to thymine upon methylation) leads to gene expression silencing. Kovalenko et al. conducted a study in which the methylation and demethylation of the *CpG* islands in the *PTENP1* 3’-UTR were targeted. They reported that the PTEN protein binds to the *PTENP1* promoter when the *CpG* islands in the *PTENP1* 3’-UTR are unmethylated, inhibiting the expression of *PTENP1-S* [110]. However, when *PTEN* is methylated, the presence or absence of the PTEN protein affects the situation. When PTEN is present, the expression of *PTENP1-S* increases; in its absence, methylation leads to the inhibition of *PTENP1-S* expression. This relationship can be exploited in treating certain cancers, such as glioblastoma cells, by making *PTENP1* a target for PTEN [110]. Overexpression of the *PTENP1* 3’-UTR led to increased levels of *PTENP1*, thereby isolating *miRNA*s and countering the PI3K/AKT pathway, reducing cell proliferation and metastatic tumors and increasing programmed cell death in the prostate (*DU145*) [28], kidney (*ACHN* and *SN12MP6*) [112], liver (*SK-Hep1* and *SMMC-7721*) [114], and breast (*MCF-7* and *MDA-MB-231*) [115, 116], bladder (*T24* and *T5637*) [117], gastric (*MGC803* and *BGC823*) [33], esophageal (*Eca19*), cervical (*CasKi* and *HeLa*) [118], and endometrial (*RL-952*,* JEC*, and *HEC-1B*) cancer cell lines [62]. These findings indicate that *PTENP1* is an important gene for regulating PTEN. However, greater complexity *was observed*,* as PTENP1 overexpression* in esophageal squamous cell carcinoma cells increased PTEN levels in *Eca19* cells but not in *TE-1* cells [119].

In breast cancer, PTEN levels are related to the presence or absence of estrogen receptors (ERs) in cells (often, hormone binding to its receptor causes cell growth) [120]. When *PTENP1* was overexpressed in ER-negative cancer cells (*MDA-MB-231/C3HBA*), it increased PTEN expression and inhibited tumor progression. Conversely, *PTENP1* overexpression in ER-positive cancer cells (*MCF-7/T-47D*) led to decreased PTEN expression and tumor growth in these cells [115, 116, 120, 121]. Therefore, both *PTENP1* and *miR*-200c are involved in decreasing PTEN levels and tumor growth in breast and endometrial cancer [2, 120, 121].

#### LncRNA-based therapeutic strategies

##### Introductory overview

Various methods have been employed to target *lncRNAs*, such as the use of small interfering RNA (*siRNA*) molecules or *CRISPR-based* techniques [36].

**Fig. 7:**
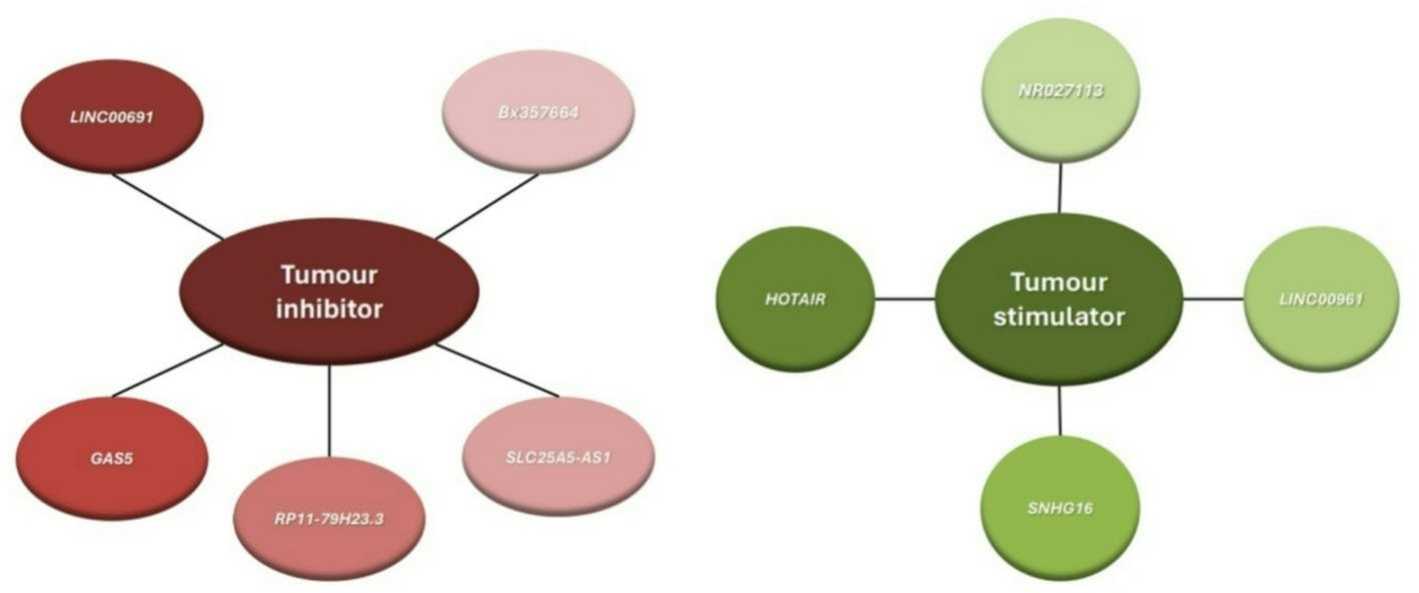
*LncRNA* types. Illustrates the *lncRNAs* presented in the previous table, which are classified into two functional groups: tumor inhibitors and tumor stimulators

Many types of *lncRNAs* act as tumor suppressors by significantly affecting the aforementioned pathway and by isolating specific *miRNAs*, thereby controlling *the* abundance and increasing the level of PTEN [122] (*see* Table 4). Dysregulation of *lncRNAs* is associated with the occurrence of various types of cancers, such as prostate, colon, lung, and breast cancers [36]. On the other hand, classifying long noncoding RNAs *(lncRNAs)* as either tumor suppressors or activators is complex due to the existence of some *lncRNA* types that activate tumors while others inhibit them [39]. Therefore, several types of *lncRNAs* and their mechanisms in tumor formation or inhibition through interactions with PTEN are discussed (see Table 4) and shown in Fig. 7.

As previously mentioned and on the basis of recent studies, *lncRNAs* can be either oncogenic or tumor-suppressive depending on the type and location of the tumor. The expression levels of different types of *lncRNAs* also vary across different tumors. Finally, many recent studies have focused on the use of *lncRNAs* to control the abundance of PTEN by acting as sponges for *miRNAs*, thereby inhibiting tumor growth (*see* Table 4), as shown in Fig. 7.

**Table 4:** Table showing the 4 types of *LncRNAs* and their mechanisms in suppressing or promoting cell growth

LncRNAs	LncRNAs mechanism of action in cancer types	Refs.	RRRR
*Bx357664*	Plays an important role in suppressing cancer formation by halting the cell cycle in the G0/G1 phase and reducing cell invasion and migration. By inhibiting the PI3K/AKT pathway by competing with *miR*-*183x-3p* as a ceRNA, and its expression has been observed to decrease in cancer cells, particularly in gastric Cancer	[36, 123]	(RRE)
*SLC25A5-AS1*	Studies have shown that reduced levels of *SLC25A5-AS1* are associated with enhanced cell growth and metastasis in various cancers. Increasing its levels is accompanied by tumor growth inhibition through halting the cell cycle in the G1/G0 phase, thereby promoting programmed cell death	[124]	
*NR027113*	Promotes tumor growth by suppressing PTEN and plays critical roles in cancer biology through cell migration and metastasis, as well as epithelial–mesenchymal transition. Its inhibition in hepatocellular carcinoma has been significant in suppressing tumor growth and reducing PI3K/AKT pathway activity	[125]	
*LINC00961*	Plays an important role in tumor development by suppressing PTEN protein and activating the PI3K/AKT pathway	[126]	
*SNHG16*	Plays an important role in tumor development by suppressing PTEN protein and activating the PI3K/AKT pathway	[127]	
*RP11-79H23.3*	Studies have shown that decreased levels are associated with a significant reduction in PTEN levels and enhanced cell growth, as observed in bladder cancer. It inhibits the PI3K/AKT pathway through its interaction with *miR*-107	[128]	
*GAS5*	Inhibits the growth of sarcoma cells by suppressing the PI3K/AKT pathway and increasing PTEN levels	[129]	
*LINC00691*	Studies have shown its positive effect on increasing PTEN protein levels and inhibiting tumor growth; By inhibiting *miR*-9-5p, which promotes tumor Formation	[130]	
*HOTAIR*	Promotes cell growth by affecting pathways that drive cancer, such as the PI3K/AKT pathway, and regulates epithelial–mesenchymal transition in breast cancer by inhibiting both PTEN and *miR*-29	[131]	

### Restoring PTEN levels via delivery of MiRNA sponges such as PTENP1/lncRNA

#### Rod-shaped viruses or nanovesicles containing PTENP1

**Fig. 8:**
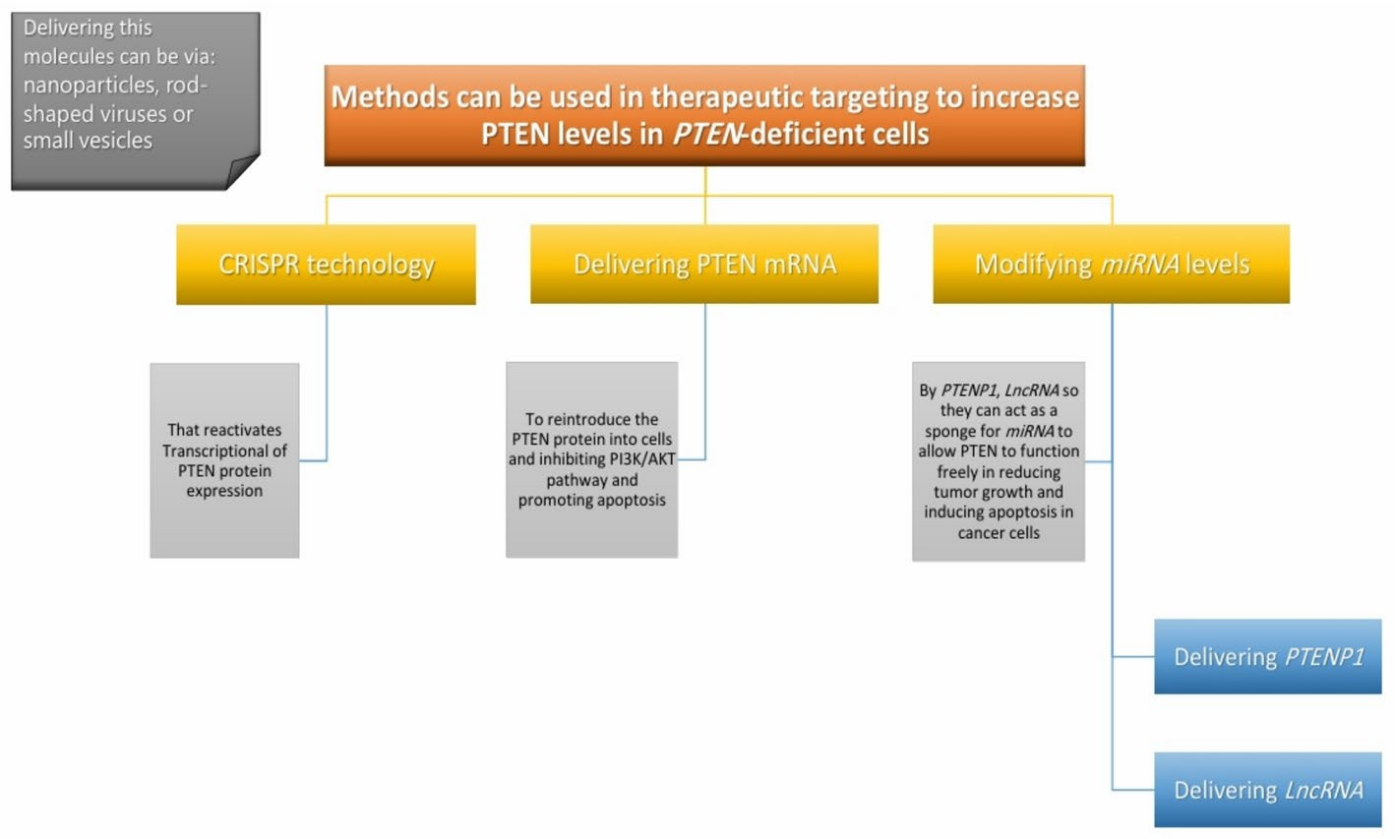
Therapeutic PTEN restoration: CRISPR technology, mRNA delivery and miRNA modulation. Illustrates therapeutic strategies to increase PTEN levels in PTEN-deficient cells, including gene targeting via CRISPR technology, vesicle-mediated delivery of PTEN mRNA or modulation of PTEN-targeting *miRNA* via delivery of *PTENP1* or *LncRNA*

PTEN mRNA levels can also be indirectly increased by delivering *PTENP1* through rod-shaped viruses or small vesicles to act as sponges for *miRNA*s, thereby allowing PTEN to function freely in reducing tumor growth and inducing apoptosis in cancer cells [132]. Several tests have been conducted, including injecting rod-shaped viruses carrying *PTENP1* into hepatocellular carcinoma (*HCC*) cells, which led to increased *PTENP1* levels in these cells [132]. The same viruses were injected into mice with *HCC*, resulting in reduced tumor growth and cell proliferation, along with the initiation of apoptosis and autophagy, thereby inhibiting *HCC* cell characteristics [132]. Moreover, *PTENP1* is transferred via small vesicles from normal cells to bladder cancer cells both in vitro and in vivo, with positive results indicating decreased tumor progression [133]. These vesicles (which carry *PTENP1*) are also delivered to glioblastoma cells (*U87MG*), where they act as sponges for *miR*-10a-5p, stabilize PTEN levels, and prevent its competitive suppression [134] (Fig. 8). The use of PTENP1 to sponge microRNAs that suppress PTEN expression has therapeutic promise but also presents notable challenges. Despite advances in their engineering, viral vesicles, which are commonly used for delivery, can trigger immune responses or lead to unintended genetic alterations [135]. There is also the issue of specificity; these vesicles may reach nontarget cells, potentially disrupting normal cellular functions [136]. Producing and storing delivery systems such as nanovesicles or viral constructs is technically demanding and requires strict conditions to maintain stability and efficacy [137]. Systemic administration adds another layer of complexity, as controlling the dose and ensuring targeted distribution remains difficult, often leading to accumulation in undesired tissues [138]. Moreover, responses to PTENP1-based strategies can differ significantly between cancer types, limiting the generalizability of this approach across tumors [139].

#### The use of various delivery methods to reduce MiRNA levels

Modifying *miRNA* levels to target both PTEN and *PTENP1* is one of the most promising therapeutic approaches for various diseases, especially cancer [140]. Increased PTEN expression is linked to increased *PTENP1* expression and decreased *miRNA* expression, which is precisely the goal. To achieve this goal, *miRNA* inhibitors known as sponges have been utilized to increase PTEN and *PTENP1* levels in cells [140]. For example, inbladder cancer, *miR*-107 is targeted via the long noncoding *RNA RP11-79h23.3*, which acts as a sponge, leading to the positive regulation of both PTEN and *PTENP1* [140]. Similarly, in endometrial cancer, *miR*-205-5p is inhibited by the long noncoding RNA *LA16C-313D11.11*, resulting in the same positive regulation [141]. In non-small cell lung cancer (*NSCLC*), *miR*-21 is targeted *via the lncRNA GAS5* to increase PTEN expression, with additional support from *the lncRNA FER1L4*, which inhibits cell proliferation and promotes apoptosis [142, 143] (see Fig. 8). The use of long noncoding RNAs (lncRNAs) to sponge microRNAs and thereby upregulate PTEN expression presents several significant challenges.

The genetic interactions between microRNAs and lncRNAs are highly complex and can vary depending on the specific cell type and its environment [144]. Efficient delivery of lncRNAs into target cells remains a major obstacle, limiting their therapeutic potential [145]. Moreover, microRNAs typically regulate multiple targets, so their inhibition through lncRNA sponging may lead to unintended off-target effects and unpredictable side effects [146]. Controlling the intracellular dosage of lncRNAs is also difficult, complicating efforts to achieve precise regulation [147]. Finally, while much of the existing research is confined to in vitro or animal models, the long-term clinical efficacy and safety of this approach in humans remain unclear [148].

Manipulating PTEN, *microRNA*, and *PTENP1* levels through therapeutic targeting represents a new frontier in cancer treatment, suggesting the potential to treat various cancer types if precise therapeutic targeting successfully surpasses the first phase of clinical trials in humans. However, one of the most significant challenges facing these therapeutic approaches is achieving precise and targeted delivery, which remains a major limitation. In addition, immune responses to introduced RNA molecules pose serious concerns that could undermine both safety and efficacy. Translating results from animal models to human applications is also difficult, largely owing to fundamental differences in genetic and cellular environments. Moreover, the lack of long-term data regarding potential side effects or overall safety continues to hinder clinical progress. Finally, the molecular complexity of the tumor microenvironment makes full control over these regulatory pathways nearly impossible at this stage.

Resistance to PTEN-targeted therapies arises through a variety of molecular mechanisms, including posttranscriptional silencing by oncogenic microRNAs, as well as activation of compensatory pathways and genetic alterations in downstream effectors. Among these mutations, activating mutations in components downstream of PTEN, particularly in PIK3CA (Q859, W780) and AKT (E17K, Q79K), have emerged as critical drivers of resistance to PI3K-targeted therapies. These alterations can sustain oncogenic PI3K/Akt signaling independently of PTEN regulation, rendering upstream interventions insufficient to suppress tumor progression [149]. In parallel, tumors frequently adapt by engaging alternative survival pathways. One prominent mechanism involves crossactivation of the MAPK/ERK cascade, which can be triggered following PI3K or Akt inhibition, thereby circumventing targeted blockade and promoting continued proliferation [150].

Moreover, reactivation of mTOR signaling, even under conditions of effective AKT inhibition, has been shown to result from both genetic rewiring and metabolic adaptation. This decoupling of mTOR from upstream AKT regulation enables persistent downstream signaling and supports therapeutic escape [151].

## Therapeutic strategies targeting PTEN loss in cancer: from akt/mtor Inhibition to extracellular vesicle-based approaches

As previously discussed, PTEN plays a tumor-suppressive role, with numerous studies showing its association with various cancers. Therefore, recent studies have focused on finding ways to treat tumors associated with PTEN loss; for example, in a clinical trial involving 1,097 patients with castration-resistant metastatic prostate cancer, treatment with ipatasertib was associated with a higher rate of Grade 3/4 adverse events and an increased rate of treatment discontinuation compared with placebo. Discontinuation occurred in 21% of the overall population and 18% of patients with PTEN-deficient tumors, with no significant difference in toxicity severity between the two groups [152]. These findings suggest that PTEN status does not substantially influence overall safety. Common adverse events (including diarrhea, hyperglycemia, rash, and elevated liver enzymes) typically appear early, between 8 and 43 days after treatment initiation, underscoring the need for close monitoring during the first few weeks. Notably, the discontinuation rate among Asian patients (32%) was greater than that among non-Asian patients (18%), despite similar baseline characteristics and comparable rates of toxicity [152]. This finding points to the possibility of unaccounted factors, such as genetic differences in drug metabolism, variations in symptom tolerance, or differences in clinical management practices. Therefore, while the overall safety profile of ipatasertib is consistent with its known effects, the higher discontinuation rate in Asian patients warrants further investigation and early supportive strategies to improve treatment adherence. This study revealed that patients with castration-resistant metastatic prostate cancer benefit from treatment with the drug ipatasertib in combination with abiraterone acetate (an AKT inhibitor), leading to tumor suppression [152]. Additionally, the use of temsirolimus (an mTOR inhibitor) was associated with tumor suppression and increased PTEN levels in mouse models [153]. On the other hand, in patients suffering from melanoma (a type of skin cancer) treated with PD0325901, the loss of PTEN was associated with a poor response of melanoma cells treated with the GSI RO4929097 (a gamma-secretase inhibitor). Finally, on the basis of recent studies, the use of PTEN could serve as a therapeutic strategy. The process of extracellular vesicle export involves bundles of lipid bilayers secreted by living cells to communicate proteins and other substances between cells, thereby controlling their function and behavior), which relies on the NEDD4 family interacting protein 1 (NDFIP1) [154].

Recent advances include the use of lipid nanoparticle-based gene therapy to restore PTEN expression in drug-resistant melanoma. For example, a novel combination of a PTEN plasmid (PL‑NANO) along with a BRD4-targeting PROTAC (AL‑NANO) resulted in synergistic tumor inhibition and apoptosis in 2D and 3D melanoma models [155].

Crucially, this strategy led to significant upregulation of PTEN and c‑Myc downregulation in BRAFi-resistant cells [155].

## Conclusion

In conclusion, this study revealed that the PTEN protein plays a crucial role in inhibiting tumor growth by suppressing pathways that contribute to its promotion, such as the PI3K/AKT pathway. We assume that integrating the analysis of EV-associated PTEN (both the protein and its regulatory RNAs) with other EV cargoes via advanced proteomics and transcriptomics could significantly improve the utility of liquid biopsies. This approach could support early detection of PTEN-deficient or PTEN-restored tumors, a more accurate prognosis by linking EV-derived PTEN levels to tumor stage or immune activity, and therapeutic monitoring, where dynamic changes in EV-PTEN could indicate treatment response or resistance. Whilereactivating the transcription of the PTEN protein can be achieved via CRISPR technology, we believe that EVs may have therapeutic implications by engineering vesicles that mimic naturally occurring EVs containing PTEN or lncRNA/PTENP1, which restore PTEN expression in target cells and suppress tumor growth. Examples include nanoparticles containing PTEN mRNA, which directly restore PTEN levels in the cell, and rod-shaped viruses or nanovesicles containing *PTENP1/lncRNA*, which indirectly increase PTEN mRNA levels by acting as sponges for miRNAs, thereby resulting in tumor suppression. These results represent an important step toward the development of new therapeutic strategies to combat cancer, opening new avenues for further research in this field; however, our understanding of the posttranscriptional regulation of PTEN (particularly its interactions with miRNAs and lncRNAs) is still limited. However, further investigations are needed to clarify these complex mechanisms. Nevertheless, upcoming clinical trials involving the delivery of miRNAs and/or PTEN and PTENP1 transcripts could offer more precise and effective treatment options for a wide range of PTEN-related cancers. Therefore, this review encourages researchers to continue exploring this vital area and to address the questions that remain unanswered.

## Data Availability

No datasets were generated or analysed during the current study.

## Abbreviations

AAV9: An adeno-associated virus 9
BRD4: Bromodomain-containing protein 4
BRAFi: BRAF inhibitors
BRRS: Bannayan–Riley–Ruvalcaba syndrome
Cas9: CRISPR-associated protein 9
CCL2: CC motif Chemokine Ligand 2
ceRNAs: competitive endogenous RNAs
c‑Myc: Cellular Myc
CpGs: Cytosine-phosphorothioate-guanine oligodeoxynucleotides
CRISPR: Clustered Regularly Interspaced Short palindromic Repeats
CTCs: Circulating tumor cells
ctDNA: Circulating tumor DNA
dCas9: dead CRISPR-associated protein 9
DDP: Cisplatin
DOX: Doxorubicin
ER: Estrogen receptor
ERK: Extracellular signal-regulated kinase
EVs: Extracellular vesicles
FU-5: Fluorouracil-5
GSI: Gamma-secretase inhibitor
GSK3: glycogen synthase kinase-3
HCC: Hepatocellular carcinoma
ILK: Integrin-linked kinase
ILVs: Intraluminal vesicles
lncRNA: long noncoding RNA
JAK: Janus kinase
MAPK: Mitogen-Activated Protein Kinase
MDSCs: Myeloid-derived suppressorcells
miRNA: microRNA
mTOR: Mammalian target of rapamycin
MVBs: multivesicular bodies
Ndfip1: Nedd4 Family Interacting Protein 1
Nedd4: Neural Precursor Cell Expressed Developmentally Downregulated Protein 4
NSCLC: Non-small cell lung cancer
PDK1: Phosphoinositide-dependent kinase-1
PDK2: Phosphoinositide-dependent kinase-2
PD-L1: Programmed death-ligand 1
PEST: Regions enriched in proline (P), glutamic acid (E), serine (S) and threonine (T)
PHTS: PTEN hamartoma tumor syndrome
PI3K: Phosphoinositide 3-kinase
PIP2: Phosphatidylinositol 4,5-bisphosphate
PIP3: Phosphatidylinositol (3,4,5)-trisphosphate
PKB: Protein kinase B
PTEN: Phosphatase and tensin homolog on chromosome 10
PTENP1: Phosphatase and tensin homolog pseudogene 1
PTENP1-AS: Phosphatase and Tensin Homolog Pseudogene 1 – AntiSense
PTENP1-AS-α: Phosphatase and Tensin Homolog Pseudogene 1 - AntiSense-α
PTENP1-AS-β: Phosphatase and Tensin Homolog Pseudogene 1 - AntiSense –β
PTENP1-S: Phosphatase and Tensin Homolog Pseudogene 1 – Sense
RTKs: receptor tyrosine kinases
SER-473: Serine 473
sgRNAs: single-guide RNAs
siRNA: small 599 interfering RNA
ssDNA: Single-stranded DNA virus
STAT: Stata
TFEB: Transcription factor EB
THR-308: Threonine 308
Tregs: Regulatory T cells
TYR: Tyrosin
3' UTR: 3′ untranslated region
VPR: VP64, p65, and Rta
